# Self-sensing intelligent microrobots for noninvasive and wireless monitoring systems

**DOI:** 10.1038/s41378-023-00574-4

**Published:** 2023-08-09

**Authors:** Zhongyi Li, Kun Wang, Chaojian Hou, Chunyang Li, Fanqing Zhang, Wu Ren, Lixin Dong, Jing Zhao

**Affiliations:** 1 School of Mechatronical Engineering, Beijing Institute of Technology, 100081 Beijing, China; 2 Beijing Advanced Innovation Center for Intelligent Robots and Systems, Beijing Institute of Technology, 100081 Beijing, China; 3 Department of Biomedical Engineering, City University of Hong Kong, 999077 Kowloon Tong, Hong Kong China; 4 School of Integrated Circuits and Electronics, Beijing Institute of Technology, 100081 Beijing, China

**Keywords:** Engineering, Physics

## Abstract

Microrobots have garnered tremendous attention due to their small size, flexible movement, and potential for various in situ treatments. However, functional modification of microrobots has become crucial for their interaction with the environment, except for precise motion control. Here, a novel artificial intelligence (AI) microrobot is designed that can respond to changes in the external environment without an onboard energy supply and transmit signals wirelessly in real time. The AI microrobot can cooperate with external electromagnetic imaging equipment and enhance the local radiofrequency (RF) magnetic field to achieve a large penetration sensing depth and a high spatial resolution. The working ranges are determined by the structure of the sensor circuit, and the corresponding enhancement effect can be modulated by the conductivity and permittivity of the surrounding environment, reaching ~560 times at most. Under the control of an external magnetic field, the magnetic tail can actuate the microrobotic agent to move accurately, with great potential to realize in situ monitoring in different places in the human body, almost noninvasively, especially around potential diseases, which is of great significance for early disease discovery and accurate diagnosis. In addition, the compatible fabrication process can produce swarms of functional microrobots. The findings highlight the feasibility of the self-sensing AI microrobots for the development of in situ diagnosis or even treatment according to sensing signals.

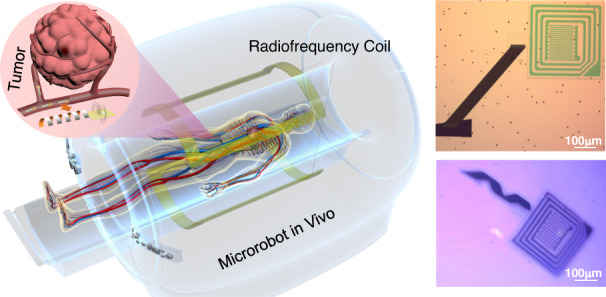

## Introduction

Microrobots have attracted considerable attention for various biomedical applications in the near future. The small size and flexible movement characteristics urge microrobots to travel minimally invasively or essentially noninvasively in narrow and complex areas for in situ diagnosis and treatment, especially targeted drug delivery, which is difficult for existing equipment^1–6^. Similar to the counterpart macrorobots, the major components for functionalizing a microrobot into a system still include perception, cognition, action, control, and more advanced intelligence^7–18^. In previous studies, researchers have focused more on how to realize precise motion control for microrobots, which relied significantly on global visual feedback and control from outside^19–28^. Embedding self-perception into microrobots remains challenging, although it possesses unique value for local diagnosis for precision medicine. Considering the remarkably elevated concentration of biomarkers near the diseased tissue compared with diluted blood and other body fluids, self-sensing microrobots can be sent almost noninvasively to concerned positions. This can be beneficial for disease diagnosis, especially during the early stage. In addition to the fixed-point detection of the target position, microrobots can patrol the body to find abnormal situations in time. Furthermore, such functionalized microrobots can be used for in situ disease mapping, continuously monitoring the changes in a microenvironment, which is of great significance for the development of therapeutic approaches. Fluorescence sensing, an effective high-resolution sensing method, has been combined with microrobots for the detection of subcutaneous, intraocular, and superficial surfaces of some organs^21,29,30^. However, single-photon excitation (ultraviolet range) can only provide a tissue interrogation depth of less than 0.5 mm; in contrast, multiphoton excitation (near-infrared range) can achieve a higher penetration rate, which is still less than 1.5–2 mm^31^. As a result, it is necessary to develop a new sensing method for microrobots to fulfill the requests for precise perception and real-time signal reading in vivo.

**Fig. 1:**
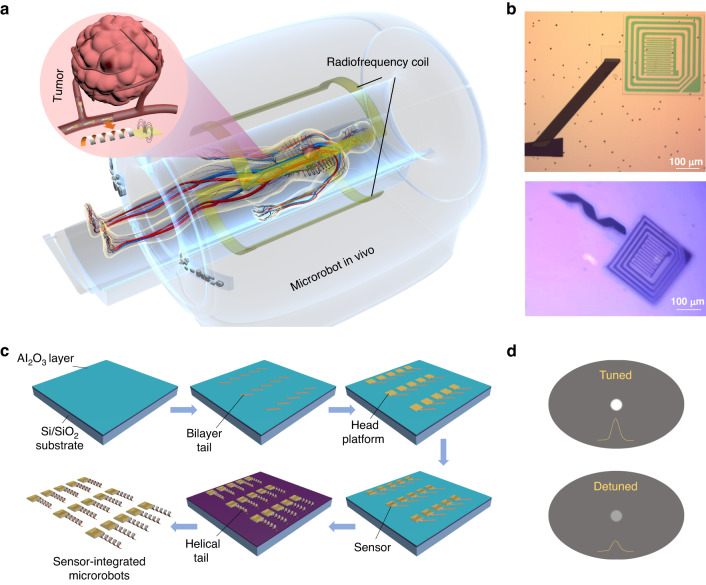
AI microrobot cooperating with electromagnetic imaging equipment for disease diagnosis. **a** Schematic diagram of AI microrobots patrolling inside the human body. The sense coil at the head of the microrobot harvested energy from the radiofrequency coil of imaging equipment and modulated the local radiofrequency magnetic field. The enhanced signal affected by the abnormal environment around the lesion suggested possible disease. **b** Microscope image of the microrobot before (top) and after (bottom) release. **c** Schematic image of the AI microrobot fabrication process. The tail, head, and sensing parts of the microrobot were fabricated sequentially before etching the sacrificial layer to release the microrobot. **d** Sketch map of contrast electromagnetic imaging under different external environments

In this paper, we propose a wireless self-sensing artificial intelligence (AI) microrobot based on local magnetic field enhancement in electromagnetic imaging for noninvasive monitoring in vivo. Our functionalized microrobot consists of a head equipped with a sensor working as a tunable radiofrequency (RF) coil and a magnetic tail that can be driven by an external magnetic field. Considering the small size of the microrobot, the thin-film-electrode sensing device is manufactured as an inductor-capacitor circuit connected by through holes to enhance local signals in the electromagnetic imaging system and transmit signals wirelessly without supplying onboard power. Various sensor circuit structures have different working ranges according to the various electromagnetism characteristics, which can be affected by the surrounding environment. With increasing size and number of pairs of interdigital electrodes, the resonance frequency of the sensing circuit decreases. Moreover, the improved permittivity around the microrobot leads to an increase in the capacitance, causing a decrease in the resonant frequency of the sensor circuit. In contrast, an increase in environmental conductivity leads to less enhancement to the local RF field by increasing the resistance of the sensor circuit. Therefore, we can obtain changed environmental signals in real time to monitor various biochemical parameters. In particular, the variable pH and temperature of the solutions can effectively influence the resistance and resonance frequency of the sensor circuit, respectively, resulting in a higher conductivity of the acid solution with a lower pH value and a lower permittivity of the solution with a higher temperature. In addition, when the resonance frequency of the sensor is tuned to the operating frequency of the imaging device, bright spots appear in the position of the microrobot in the image, as shown in Fig. 1d. The novel self-sensing AI microrobot, with the potential to benefit from the advantages of existing electromagnetic imaging equipment in transmitting signals in the human body, provides a new route to realize passive wireless sensing in various complex positions, further simplifying the sensor structure and reducing the size.

## Results and discussion

**Fig. 2:**
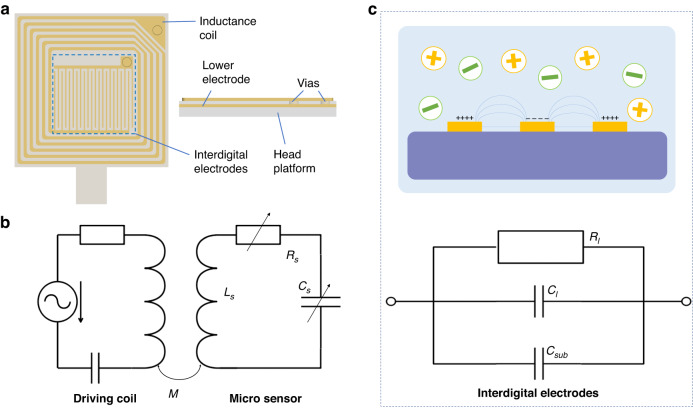
Operating principle of the wireless sensor. **a** Top and front views of the wireless sensor. The connected external coil and internal interdigital electrodes form an RLC circuit through the via holes. **b** Working principle diagram of the wireless sensor. The sensor was excited by the external electromagnetic signal from the coupled driving coil. ${R}_{s}$, ${L}_{s}$ and ${C}_{s}$ represent the sensing resistance, inductance, and capacitance of the sensor, respectively. **c** Schematic image and equivalent circuit of interdigital electrodes, in which ${R}_{l}$ and ${C}_{l}$ are the solution capacitance and solution resistance, respectively, while$\,{C}_{{sub}}$ is the substrate capacitance

Figure 1a schematically shows the scenario of the AI microrobot patrolling inside the human body under external control. Among the existing electromagnetic imaging methods, magnetic resonance imaging (MRI) has been widely used because of its high penetration depth and spatial resolution. During MRI imaging, the imaging signal strength is positively correlated with sin(α), in which α is the flip angle of the excitation pulse. The strength of the electromagnetic field affects the angle during imaging and further determines the final imaging signal^32^. The sensing part at the head of the microrobot harvested the energy from the electromagnetic field generated by the RF coil of the imaging device and modulated the local RF magnetic field to change the signals at the corresponding position in the image^33,34^. The dielectric characteristics of the surrounding environment, including the dielectric constant and conductivity, determine the electrical characteristics of the sensing circuit and further affect the enhancement effect on the local RF magnetic field. Diseases can change the dielectric properties of the microenvironment, and therefore, when the microrobot reaches the vicinity of the lesion, the enhanced signal can be changed, suggesting the occurrence of possible disease^35,36^. Figure 1b shows a microrobot patterned on the substrate and an as-fabricated free-standing microrobot. The designed device is manufactured step by step following the processes shown in Fig. 1c. First, a 30 nm Al_2_O_3_ layer was deposited as the sacrificial layer on a preprepared Si substrate covered with 300 nm SiO_2_. Then, a double-layer tail containing a magnetic (Fe) and a prestress membrane (Si_x_N_y_) was deposited. Therein, the prestress Si_x_N_y_ membrane was utilized as the main driving force of helical tail shape formation based on 3D rolling-up technology. 3D rolling-up technology is a reliable 3D forming technology resulting from the generation of the bending moment, along with the continuous release of the prestress Si_x_N_y_ membrane. Here, a precompressive Si_x_N_y_ membrane possessed a pair of opposite forces at the release interface, enabling an upward bending moment. With the isotropic release process of the initial parallelogram mesa, the helical shape can be successively formed. Owing to the final geometric structure morphology depending on the initial release shape design, the balance between the driving force and thin film stiffness, and so on, can be obtained^37,38^. The head platform of the microrobot was made of 100 nm SiO_2_, which acted as the substrate to support the sensor. The three-layer structure of the sensor was sequentially fabricated on the head of the microrobot. The middle-interdigitated electrodes (<10 μm thickness) occupied approximately 25% of the head area and were surrounded by annular electrodes, which were connected to the bottom electrode through two via holes to form a closed resistance‒inductance-capacitance (RLC) loop, as shown in Fig. 2a. The size of the RF coil ranged from ~500 to ~50 μm, and the number of interdigital electrode pairs could be adjusted according to the actual requirements, as shown in Supplementary Fig. S2. Finally, the chip was soaked in NaOH solution to etch the sacrificial Al_2_O_3_ layer, and the stress in the Si_x_N_y_ layer was released, leading it to roll up into a helical tail. A more detailed fabrication process is shown in Supplementary Fig. S1.

Figure 2b demonstrates the principle diagram of the RLC passive sensor. ${L}_{s}$, represents the inductance introduced by the coil. ${R}_{s}$ and ${C}_{s}$ represent the sensing resistance and capacitance of the sensor introduced by interdigitated electrodes. The driving coil of the imaging equipment first generates the RF magnetic field, and the external loop coil of the sensor harvests the energy from the RF magnetic field and induces the current of the sensor circuit. The corresponding current can excite an additional magnetic field, which enhances the local RF magnetic field of the sensor. Considering that the distance between the excited coil and the sensor is far enough, the coupling coefficient is usually very small, resulting in the influence of the magnetic field generated by the sensor on the excited coil being negligible. Therefore, the excited magnetic field around the sensor, as well as the voltage generated by the sensor coil, can be considered a constant. As a result, the current and magnetic field of the sensor merely depend on the impedance of the circuit, and the sensor circuit can obtain the maximum current and the most obvious enhancement effect on the local RF magnetic field at the resonance frequency. The resonance frequency (${f}_{s}$) and quality factor (${Q}_{s}$) can be calculated using the following equations:

1 $${f}_{s}=\frac{1}{2\pi \sqrt{{L}_{s}{C}_{s}}},{Q}_{s}=\frac{1}{{R}_{s}}\sqrt{\frac{{L}_{s}}{{C}_{s}}}$$

where ${L}_{s}$ is a constant. ${C}_{s}$ and ${R}_{s}$, introduced by interdigitated electrodes, can change with the surrounding environment, as shown in Fig. 2c (top). When the dielectric and conductive properties of the surrounding solution changed, the equivalent resistance and capacitance of interdigital electrodes responded accordingly. In the equivalent circuit in Fig. 2c (bottom), ${R}_{l}$ and ${C}_{l}$ represent the resistance and capacitance when the electric field passes through the solution, respectively, while ${C}_{{sub}}$ represents the substrate capacitance when the electric field passes through the substrate. The total capacitance ${C}_{{IDE}}$ is the sum of ${C}_{l}$ and ${{C}}_{{sub}}$_._ The equivalent impedance *Z* can be calculated using the following formulas:

2 $$C={C}_{{IDE}}$$

3 $$R={R}_{l}$$

4 $$Z=\frac{1}{{\rm{j}}{\rm{\omega }}{\rm{C}}}//\text{R}=\frac{1}{{\rm{j}}{\rm{\omega }}{\rm{C}}+1/{\rm{R}}}=\frac{1/{\rm{R}}-{\rm{j}}{\rm{\omega }}{\rm{C}}}{{{\rm{\omega }}}^{2}{{\rm{C}}}^{2}+1/{{\rm{R}}}^{2}}$$

and ${R}_{s}$ and ${C}_{s}$ in the sensor circuit can be calculated as follows:

5 $${R}_{s}=\mathrm{Re}({R}_{l}//{C}_{{IDE}})=\frac{1/R}{1/{R}^{2}+{\omega }^{2}{C}^{2}}=\frac{1}{1/R+{\omega }^{2}{C}^{2}R}$$

6 $$\omega {C}_{s}=-\frac{1}{{Im}({R}_{l}//{C}_{{IDE}})}=\frac{1/{R}^{2}+{\omega }^{2}{C}^{2}}{\omega C}=\frac{1}{{R}^{2}\omega C}+\omega C$$

in which ${R}_{s}$ decreases with increasing $\omega C$ and $\omega {C}_{s}$ decreases with increasing $R$. ${R}_{s}$ and $\omega {C}_{s}$ obtain the maximum and minimum values at $R=\frac{1}{\omega C}$ if $\omega C$ and $R$ are fixed, respectively. When $R > \frac{1}{\omega C}$, the increase in $R$ leads to a decrease in ${R}_{s}$, and the increase in $\omega C$ leads to an increase in $\omega {C}_{s}$. In contrast, when $R < \frac{1}{\omega C}$, the increase in $R$ induces an increase in ${R}_{s}$, and the increase in $\omega C$ results in a decrease in $\omega {C}_{s}$. Especially when $R\gg \frac{1}{\omega C}$:

7 $${R}_{s}=\frac{1}{1/R+{\omega }^{2}{C}^{2}R}\approx \frac{1}{{\omega }^{2}{C}^{2}R}$$

8 $$\omega {C}_{s}=\frac{1}{{R}^{2}\omega C}+\omega C\approx \omega C,{C}_{s}\approx C$$

According to the structural model, the electrical characteristics of interdigital electrodes were emulated by a high-frequency structure simulator (HFSS). The interdigital electrode model was extracted from the microrobot with a head size of 500 μm and 11 interdigital electrode pairs. With the frequency varying from 0.5 to 2 GHz, the amplitudes of the real and imaginary components of the impedance decrease clearly, as shown in Supplementary Fig. S3. The increase in environmental conductivity will lead to an increase in the real impedance and a decrease in the imaginary impedance. Notably, when the conductivity increases from 0.001 to 0.1 S/m, the imaginary impedance is almost unchanged, which agrees with the theory of $R\gg \frac{1}{\omega C}$. The simulation results are consistent with the theoretical analysis results at $R > \frac{1}{\omega C}$. Therefore, the increased environmental conductivity can lead the loop resistance to increase at the resonance frequency and weaken the enhancement effect.

**Fig. 3:**
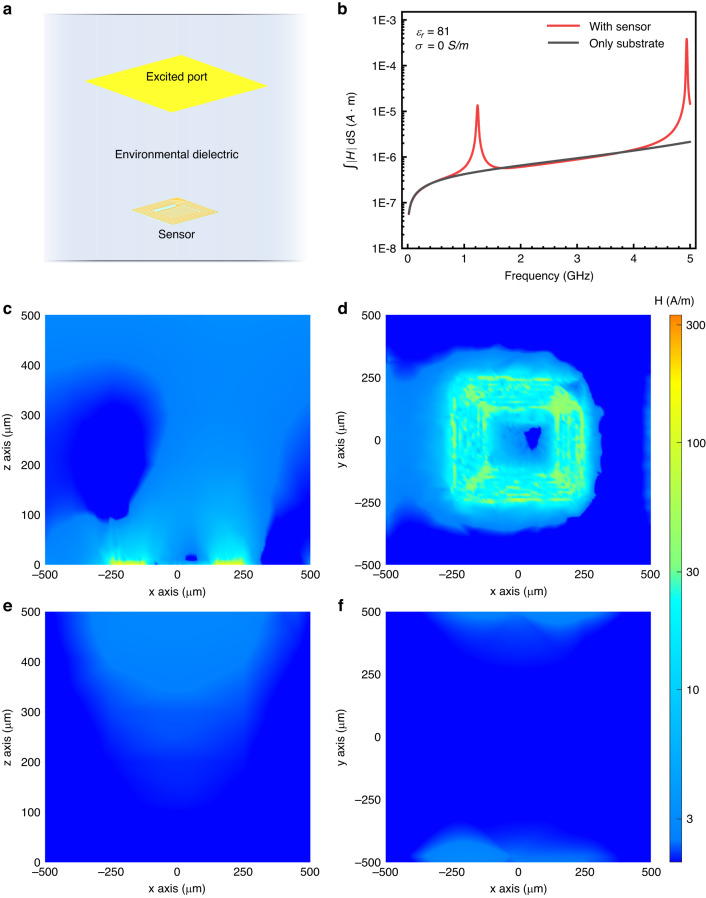
HFSS simulation of the sensor. **a** Schematic diagram of the simulation model. The sensor is surrounded by the environmental dielectric, while the top exacted port generates the electromagnetic field. **b** The simulation results of the relationship between the local magnetic field intensity and frequency. The sensor-enhanced local magnetic field intensity will reach the maximum value at the resonance point. **c**, **d** The side and top views of the electromagnetic field distribution around the sensor at the first resonance point, respectively. **e**, **f** The contrast electromagnetic field distribution without a sensor at the first resonance point

The local electromagnetic field enhancement ability of the sandwich structure sensor, including the intermediate dielectric layer and top and bottom electrodes, can be simulated by the HFSS, as shown in Fig. 3a, in which an excited port is used to generate the RF magnetic field and the sensor surrounded by the dielectric as the environmental substitution is placed below the excited port. The substrate without the sensor is set for the control group, and deionized (DI) water (${\varepsilon }_{r}=81,\sigma =0\text{S}/\text{m}$) is used as the environmental dielectric. The local RF magnetic field strength can be evaluated by magnetic field strength integration. In Fig. 3b, the $500\times 500\,{{\rm{\mu }}{\rm{m}}}^{2}$ plane centered on the upper electrode is used to calculate the magnetic field strength. As the frequency increases, the strength integration of only the substrate model increases gradually. In contrast, some definite peaks appear for the sensor, illustrating the enhanced local RF magnetic field effect. According to the theoretical analysis, the greatest enhancement effect occurs at the resonance frequency, the first of which is ~1.24 GHz. The magnetic field strength integral for the substrate is ~4.8E-7 T ∙ m^2^ at 1.24 GHz, while the value can amplify ~45 times to ~2.2E-5 T ∙ m^2^ for the sensor. In addition, the enhancement amplitude reaches ~560 times at the second resonance point (f = 4.94 GHz). Moreover, the presence of the sensor greatly changed the local magnetic field distribution, especially in the area close to the sensor. The local RF magnetic field distribution of the sensor at 1.24 GHz is shown in Fig. 3c, d. The maximum electromagnetic field strength can reach ~300 A/m at 1.24 GHz. With increasing distance from the sensor, the corresponding strength decreases gradually. However, the maximum magnetic field strength is only ~3 A/m without the sensor, as shown in Fig. 3e, f. The magnetic field in the central area around the substrate is uniform and less than 1 A/m, while the magnetic field strength becomes larger at the boundary and the area closer to the excited port.

The magnetic field enhancement coefficient (MFEC), the ratio of the magnetic field strength integral with and without the sensor, can be utilized to evaluate the magnetic field enhancement effect at different planes, as shown in Eq. (9).

9 $${MFEC}=\frac{{\iint }_{D}\left|{H}_{{sensor}}\right|{dS}}{{\iint }_{D}\left|{H}_{{substrate}}\right|{dS}}$$

The size of the plane is $500\times 500\,\mu m$, which is similar to the sensor at the center, and the height gradually increases from the upper substrate plane ($D=\,0$ μm), where the MFEC is ~45.45. As the height increases, the relevant value of the MFEC decreases accordingly. When $D=\,200$ μm, the MFEC changes to ~1.19. When D increases further to ~300 μm, the MFEC decreases to ~1.03, but the ability to change the magnetic field distribution remains strong according to the magnetic field distribution in Supplementary Fig. S4.

The sensor structure, including the size of the sensor and the number of interdigital electrode pairs, can affect the magnetic field strength, as shown in Supplementary Fig. S5. We compared the MFEC at $D=0$ μm with different sensor structures. The whole microrobot swam in the DI water. According to the simulation results, the resonance frequency peak increased rapidly from ~1 to ~30 GHz as the size of the sensor decreased from 500 to 50 μm. Moreover, the frequency peak has a left shift with increasing numbers of interdigital electrode pairs, which can promote the capacitance and reduce the resonance frequency. The statistical result of the shift in the resonance peak with the change in sensor structure is summarized in Supplementary Fig. S6. Overall, the small size of the sensor led to a high resonance frequency, resulting from the limitation of the placement space leading to smaller values of inductance and capacitance.

**Fig. 4:**
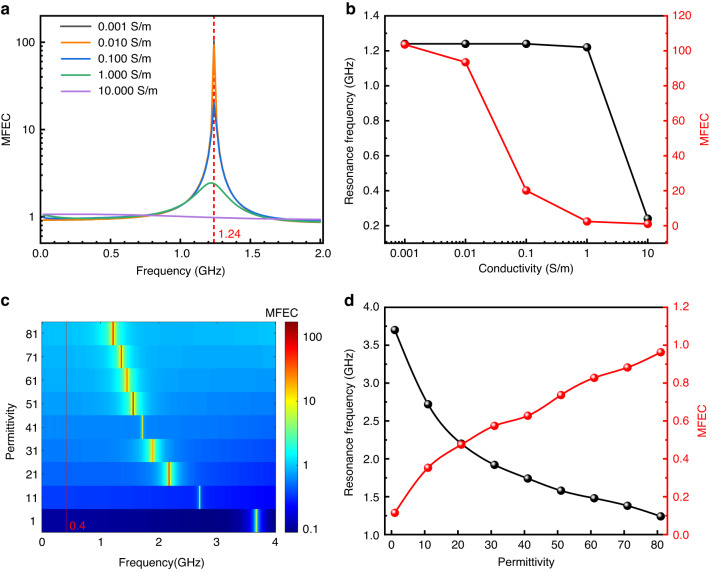
Simulation of device performance depends on the conductivity and permittivity of the environmental dielectric. **a** Relatively enhanced coefficient of local radiofrequency magnetic strength (MFEC) as a function of sweeping frequency. **b** The resonance frequency and MFEC as a function of conductivity. **c** Sweeping frequency test of MFEC with varying permittivity from 1 to 81. **d** The resonance frequency and MFEC as a function of the permittivity

The surrounding environment can affect the local magnetic field enhancement performance of the sensor by changing the permittivity or conductivity. With the conductivity increasing exponentially from 0.001 to 1 S/m, under a fixed permittivity (${\varepsilon }_{r}=81$), resonance peaks are concentrated at approximately 1.24 GHz when the swept frequency ranged from 0.1 to 2 GHz, as shown in Fig. 4a. The data at $D=0$ μm were used to calculate the MFEC. However, when the environmental conductivity is 10 S/m, the large resistance of the sensor circuit causes a small current, resulting in the local magnetic field remaining nearly unchanged. The relationship between the resonance frequency, the MFEC, and the conductivity is plotted in Fig. 4b based on the results from Fig. 4a. The MFEC curve was extracted at a frequency of 1.24 GHz (the red line in Fig. 4a, near the resonance peak). The stable resonance frequency with conductivity varying from 0.001 S/m to 0.1 S/m is mainly due to the small conductivity, where the solution impedance of the interdigital electrode ${R}_{l}$ is much larger than $\frac{1}{\omega {C}_{{IDE}}}$. Correspondingly, the capacitance of the whole circuit ${C}_{s}$ and the resonance frequency ${f}_{s}$ remain unchanged. When the conductivity increased to 1 S/m, the corresponding resonance frequency decreased to 1.22 GHz. Moreover, the resonance frequency dropped rapidly to 0.24 GHz when the conductivity reached 10 S/m. Overall, the MFEC is more sensitive to small conductivity changes, demonstrating a monotonic downward trend with increasing conductivity at a frequency of ~1.24 GHz. It decreased from 103.6 at 0.001 S/m to 2.4 at 1 S/m but was only 0.98 at 10 S/m, indicating that there was no enhancement effect.

Figure 4c shows the simulation results of permittivity from 1 to 81 under constant conductivity (σ = 0 S/m) with frequencies ranging from 0.1 to 4 GHz. With increasing permittivity, the resonance peak revealed a redshift from 3.7 to 1.24 GHz. Meanwhile, the MFEC was obviously improved from 0.1 to over 100 at the resonance point. The detailed corresponding relationship between resonance frequency and permittivity is plotted in Fig. 4d. The decrease rate of the resonance frequency gradually slowed down with increasing permittivity. When the permittivity changed from 1 to 11, the resonance frequency dropped from 3.7 to 2.72 GHz. However, the frequency decreased by only 0.14 GHz (from 1.38 to 1.24 GHz) under permittivity varying from 71 to 81, suggesting that the tuned sensitivity of the resonance frequency provided high detection accuracy and response under conditions with low permittivity. To cooperate with the electromagnetic imaging system, we extracted MFEC results with the different dielectrics at 400 MHz, as shown in Fig. 4d (right), corresponding to MRI with operating magnetic field strength (B_0_) of 9.4 T. Compared with the value near the resonance peak, the MFECs at 400 MHz are all less than 1, which means that the sensor weakens the local RF magnetic field instead, resulting from the induced magnetic field generated by the coil reducing the excited magnetic field. However, the induced magnetic field is large enough to amplify the field strength near the resonance peak. When the permittivity changes from 1 to 81, the MFEC linearly increases from 0.11 to 0.96. A MFEC of less than 1 can make the sensor appear as a dark spot in the electromagnetic imaging system while the surrounding environment presents as a bright background. Therefore, it is necessary to match the resonance peak with the working frequency of electromagnetic imaging to realize variation and easy observation for in vivo detection.

**Fig. 5:**
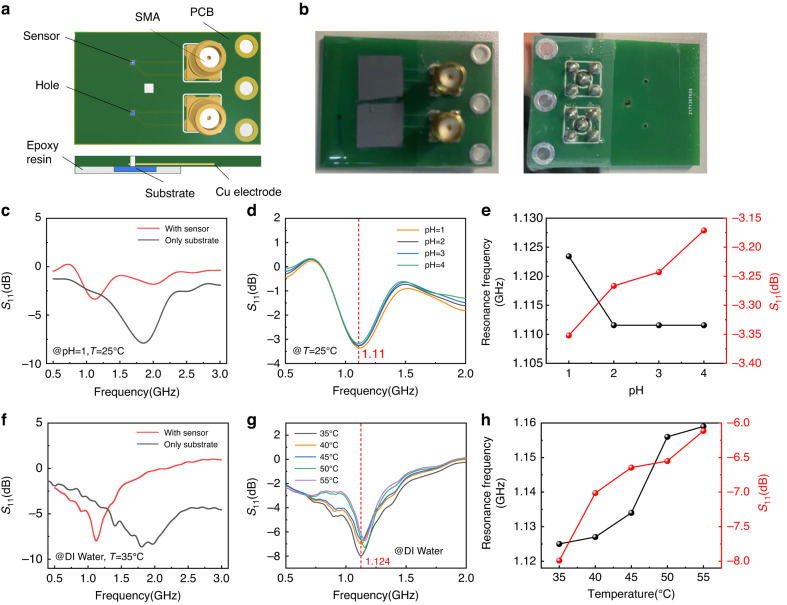
Electromagnetic characteristics measurement of the sensor using the network analyzer. **a** The model of the PCB for the sensor measurement. A single-turn coil with a length of ~1 mm was connected to the network analyzer through the Cu electrode and SMA as the external coil. The sensor was immersed in different solutions dropped through the middle hole. **b** Optical images of the PCB for measurement. **c** Compared changes in the reflection coefficient S_11_ with and without the sensor when pH = 1. **d** S_11_ as a function of the frequency with the solution pH varying from 1 to 4. **e** Response characteristics of the sensor at different pH values. **f** Compared shifts of S_11_ with and without the sensor at 35 °C. **g** S_11_ as a function of the frequency with the water temperature varying from 35 to 55 °C. **h** Response characteristics of the sensor at different temperatures

On account of the theoretical results, we fabricated a suitable structure sensor on the microrobot and connected the device to the prepared external measuring equipment, as shown in Fig. 5a. A square coil with a side length of 1 mm was used as an external readout coil, which was coupled with the coil surrounding the perceptive interdigital electrodes. The external coil was connected to the network analyzer through a Sub-Miniature-A (SMA) connector for the frequency sweeping test, and the measured circuit was made on a printed circuit board (PCB). The chip carrying the device was fixed on the PCB by epoxy resin to prevent the solution from affecting the readout coil from the back. As shown in Fig. 5b, the solution can immerse the lower end of the PCB and contact the sensor through the front hole during the measurement. The reflection coefficient S_11_, which reflects the sensor response to the surrounding environment, can be calculated by Eq. (10):

10 $${S}_{11}=20{\log }_{10}\left(\left|\frac{{Z}_{{eq}}-{Z}_{0}}{{Z}_{{eq}}+{Z}_{0}}\right|\right),{Z}_{{eq}}={R}_{r}+j\omega {L}_{r}+\frac{{\omega }^{2}{M}^{2}}{{R}_{s}+j\omega {L}_{s}+\frac{1}{j\omega {C}_{s}}}$$

${Z}_{{eq}}$ and *Z*_*0*_ are the equivalent impedances of the readout coil and the system (normally 50 Ω), respectively. *M* is the coupling coefficient of the readout and the sensor coil. *R*_*r*_ and *L*_*r*_ are the resistance and inductance of the measured circuit, respectively. *S*_*11*_ reaches the minimum value near the resonance frequency when Q_s_ ≫ 1^39^. We prepared HCl solutions with different conductivities by changing the concentration, demonstrating the relevant pH value. When the device was placed in the HCl solution at pH = 1 and T = 25 °C, the resonance frequency of the sensor centered at ~1.12 GHz, while the resonance frequency is ~1.85 GHz for the substrate only, as shown in Fig. 5c. As the pH increases from 1 to 4 in Fig. 5d, the curves shift to higher frequencies. The relationship between the resonance frequency and the pH is plotted in Fig. 5e based on the result from Fig. 5d. When the pH value decreases to 1, the resonance frequency rises to ~1.120 GHz. The free H^+^ in the solution forms the diffusion capacitance, which attenuates the external electric field, resulting in the sensor capacitance decreasing instead of increasing^40^. However, the resonance frequency remains nearly unchanged at ~1.110 GHz when the pH is close to 7 because of the low concentration of the solution, which leads to a larger resistance than the impedance generated by the capacitance. Nevertheless, the S_11_ at 1.110 GHz (the red line in Fig. 5d) as a function of pH in Fig. 5e monotonically increases from −3.35 dB to −3.17 dB when the pH increases from 1 to 4, indicating the sensitivity of the device as a pH sensor.

Moreover, the sensor can respond to ambient temperature changes. Solutions with different temperatures were obtained by heating the DI water, and the permittivity gradually decreased from ~80 at room temperature (25 °C) to less than 70 at 60 °C^41,42^. The resonance frequency moved from ~1.90 GHz to ~1.12 GHz when the device was fabricated on the substrate, which was surrounded by DI water at T = 35 °C, as shown in Fig. 5f. When the temperature increased from 35 °C to 55 °C, both the resonance frequency and the S_11_ of the device increased. Figure 5h demonstrates the relationship between the resonance frequency and the temperature derived from the measured result in Fig. 5g. The resonance frequency increases from 1.125 to 1.160 GHz linearly with increasing temperature due to the decreased permittivity, while S_11_ at 1.124 GHz monotonically changes from −7.99 to −6.11 dB, which is much larger than the change caused by the varying pH values.

Therefore, the sensor integrated into the microrobot can modulate the local magnetic field in the electromagnetic imaging process depending on the surrounding environment and affect the signals at the position in the image. Apparently, the AI microrobot can be guided to the prescribed destination and monitor the local microenvironment under the actuation magnetic field. We fabricated a series of microrobot integrated sensors with different sizes (50–500 μm) and released them into DI water, as shown in Supplementary Fig. S8. Although the smaller microrobots could move in narrow channels and complex areas of the body, their resonance frequency was higher, causing difficulties while cooperating with the electromagnetic imaging equipment.

**Fig. 6:**
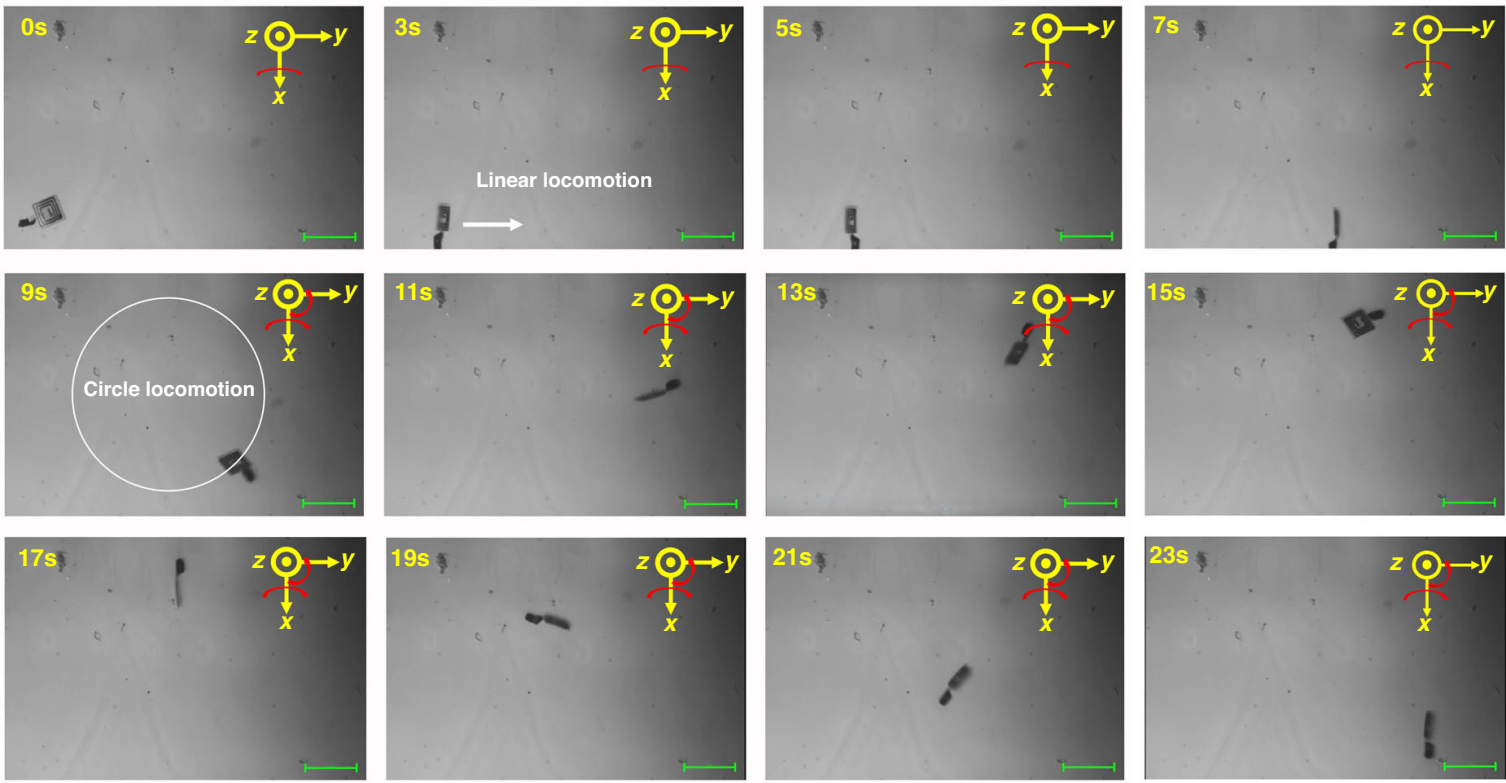
Time-resolved optical images of the locomotion control and posture adjustment of an integrated AI microrobot. Linear motion was generated in the lateral direction by 7 s, followed by a circular motion. Then, a half circle started at 7 s and finished at 15 s, and a whole circle route was completed at approximately 22 s; the scale bar is 200 μm

Locomotion control and posture adjustment of our integrated AI microrobots were carried out to attain the motion characteristics of AI microrobots, as shown in Fig. 6 and Supplementary Movie 1. The actuation magnetic field was generated by three pairs of Helmholtz coils perpendicular to each other, and the intensity and direction of the resultant rotational field could be adjusted according to the motion requirements. The tail was rotated by the external rotating magnetic field, and the rotation was converted by the helical structure into a translational one so that the microrobot could move forward and backward according to the direction of rotation of the field. DI water was utilized as the work environment. During the whole driving experiment, both linear and circular locomotion was achieved from 0 s to 7 s and from 7 s to 23 s, respectively. In the first linear locomotion stage, a rotating uniform magnetic field in the *yz* plane was applied, enabling the AI microrobot to obviously spin and crawl like an active paddle accompanied by the external magnetic field stimulus. The magnetic field is represented as follows:

11 $$\vec{{B}_{\text{yoz}}}={B}_{0}\sin (2\pi {f}_{1}t)\vec{j}+{B}_{0}\cos (2\pi {f}_{1}t)\vec{k}$$

where *B*_0_ is the magnetic induction intensity, *B*_0_ = 6 mT; and *f*_1_ is the rotation frequency, *f*_1_ = 1 Hz. By comparing the position of the microrobot at 3 s and 7 s, the linear locomotion speed reached 133.32 μm/s. This large speed was mainly attributed to a tumbling motion based on a rotation axis at the interface line between the microsensor and substrate, leading to the 100 μm length of the microsensor becoming the final rotation radius. Then, to achieve circular locomotion, a gradually increasing yaw angle with an increment speed of 24°/s in the *xy* plane was applied to the initial *B*_*yoz*_ field. The total external magnetic field is as follows:

12 $$\vec{{B}_{\text{total}}}={B}_{0}\sin (2\pi {f}_{1}t)\sin (\omega t)\vec{i}+{B}_{0}\sin (2\pi {f}_{1}t)\cos (\omega t)\vec{j}+{B}_{0}\cos (2\pi {f}_{1}t)\vec{k}$$

where *ω* is the yaw angular speed. The results showed that a circular path started at 7 s and finished at approximately 22 s. Thus, the average angular speed can be calculated as *ω* = 2π/15 = 0.419 rad/s. In addition, our integrated microrobot with a larger microsensor (300 μm length) was also successfully actuated by using the same external magnetic field, as shown in Supplementary Movie 2. The results showed that the optimized helical tails with larger geometry dimensions could offer a larger driving force to guarantee the tumbling motion of larger microsensors due to the larger magnetic material volume integrated into the helical tails. This phenomenon indicated that our manufacturing process was suitable for integrated microrobots with various scales. In conclusion, our integrated microrobot exhibited fast locomotion speed and flexible path planning. Next, the formation mechanism of tumbling of our integrated AI microrobot was examined. In our integrated platform, a continued and stable driven moment, reliably formed as a result of the magnetic torque, always needed to align the magnetization of the microrobot with the applied field. However, the unsymmetrical geometric dimensions between the microsensor and helical tails lead to the position of the applied force always being located at the interfacial line between the microsensor and the Si substrate. With the help of the rotating magnetic field, the periodic friction force at the interface between the microsensor and the Si substrate enabled the AI microrobot to transform the rolling motion into linear motion in the plane, finally forming a paddling motion. This transformation motion characteristic is similar to the tumbling motion of peanut microrobots in a previous report^23^. More importantly, due to the rotation of microsensors, the posture adjustment of our integrated AI microrobots can also be realized, enabling precise control of the relative rotation angle between the integrated coil and the external coil. This effective regulation behavior will promote the effective coupling between the coils and ensure the reliability of wireless signal extraction. Therefore, our integrated AI microrobots provide an effective integration strategy for future wireless monitoring systems with a controllable movement mode. In the future, optimized AI microrobot architectures need to be designed to achieve the conversion of helical rotational motion into linear motion, away from the substrate effect.

Our research strongly implied that the designed sensor integrated into the microrobot could realize controllable mobile sensing in vivo for disease diagnosis. The magnetic layer at the tail of the microrobot can generate force to actuate the microrobot under the excitation of an external magnetic field, and the wireless sensor can modulate the local RF magnetic field without providing external power. When the functional microrobot worked in the electromagnetic imaging system, the device position appeared as an artifact in the image, especially bright spots when the resonance frequency of the sensor matched the working frequency of the electromagnetic imaging. The conductivity and permittivity of the environment changed the modulation effect of the local RF magnetic field. Compared with in vitro detection, the abnormality around the lesion is more prominent, especially during the early stage of disease. Therefore, the designed device sent to the vicinity of the lesion can improve the detection accuracy of the disease. The AI microrobot is minute enough to enter the human body noninvasively and can realize detection in complex positions in the body. Owing to advanced micro/nanofabrication technology, large-scale parallel manufacturing can reduce cost and produce good consistency. In addition, the AI microrobot can cooperate with the electromagnetic imaging equipment to transmit the detection signal wirelessly, so the sensor itself only needs to locally interact with the RF magnetic field and modulate it. According to a previous study, the maximum working frequency of the MRI scanner for the human body is ~500 MHz under an operating magnetic field strength (B_0_) of 11.7 T^43^. To effectively reduce the resonance frequency of the AI microrobot to match the MRI, we can increase the number of interdigital electrodes or increase the thickness of the upper electrode, as shown in Supplementary Fig. S7. Moreover, the refined structural design, including optimization of the inductance coil and loading of fixed capacitance and resistance, can facilitate cooperation with the MRI equipment.

Although the designed sensor was sensitive to the conductivity and permittivity change in the surrounding environment, which was usually related to the occurrence of diseases, it is not specific to different influence factors. Next, we will functionalize the upper electrode of the sensor by coating it with selective materials to specifically detect the concentrations of various ions, proteins, glucose, oxygen, and so on^44–49^. After the functionalized sensor is combined with the marker, the permittivity and conductivity above the sensor will change. Therefore, the scene in which many microrobots, patrolling inside the body under external control, are navigated by MRI and actively find abnormal parts according to the sensing signal for in situ diagnosis and even treatment will be realized in the near future.

## Conclusion

In summary, a wireless AI microrobot designed for noninvasive in vivo monitoring was fabricated to realize self-sensing functionality. The novel AI microrobot, consisting of a head sensor and a magnetic helical tail, can perceive environmental change sensitively based on the enhanced signal in electromagnetic imaging and can be precisely driven by an external actuating field. The sensor circuit harvests energy from the RF magnetic field without onboard power and is tuned or detuned by the conductivity and permittivity of the surrounding environment. This determines the enhancement effect on the local RF magnetic field, increasing it up to ~560 times. An increase in permittivity led to a corresponding decrease in the resonance frequency, while the increase in conductivity weakened the enhancement effect. Therefore, the impedance signal, affected by the surrounding environment, can reflect the working state of the microrobot in real time. Moreover, the magnetic helical tail actuated the microrobot to the target position under the control of an external magnetic field accordingly. Considering the AI microrobot’s small size, controllable movement, and self-sensing properties, it has great potential to be used in noninvasive monitoring of the body combined with existing electromagnetic imaging equipment, especially those places that are difficult to reach by traditional equipment. Additionally, the integration of a passive sensor with the microrobot highlights the feasibility of noninvasive wireless sensing in situ, which is of great significance for the development of multiple therapeutic means. Moreover, on account of the compatible fabrication process, mass-produced and cluster-controlled microrobots are predictable. Furthermore, integrating various functional coatings on the AI microrobot can enable the detection of various biochemical components and biomarkers for specific disease diagnoses in the future^50,51^.

## Methods

### Device fabrication

The silicon substrate with a 300-nm-thick silicon dioxide film was first successively cleaned with acetone, isopropyl alcohol (IPA), and DI water. Then, a 30-nm Al_2_O_3_ film was deposited on the substrate using e-beam evaporation as a sacrificial layer. A 30-nm silicon nitride (Si_x_N_y_) membrane with compressive stress was grown with plasma-enhanced chemical vapor deposition (PECVD). Then, the magnetic helical tails were made of a Fe membrane by using UV lithography, development, metallization by magnetic sputtering and lift-off. To achieve helical tails with different dimensions, the balance between the Fe membrane and the prestrained Si_x_N_y_ needed to be optimized. Here, we used a 150-nm-thick Fe film at a sputtering deposition rate of 0.15 nm/s. Then, inductively coupled plasma (ICP) dry etching was utilized to etch the Si_x_N_y_ membrane until the Al_2_O_3_ layer was exposed, obtaining batched helical tail mesa arrays. The etching gas was CHF_3_, and the etching power was 200 W.

A 100 nm SiO_2_ layer was deposited by electron beam evaporation, and the lower electrode of the sensor (100 nm Au) was fabricated via UV lithography, development, thermal evaporation, and lift-off. Another photoresist was then patterned as a soft mask for SiO_2_ etching. The SiO_2_ layer was etched by ICP to form the head platform of the microrobot. Then, another 100 nm SiO_2_ dielectric layer was deposited by electron beam evaporation and patterned by photolithography, etching, and photoresist removal processes to serve as the intermediate dielectric layer for the sensor. The second SiO_2_ layer was etched by reactive ion etching (RIE). The etching gas was CF_4_, and the power was 150 W. The upper patterned electrodes (300 nm Au) were deposited by thermal evaporation after UV lithography.

Finally, the sample was immersed in a 45% sodium hydroxide solution to etch the Al_2_O_3_ layer. Both the microsensors and helical tails were separated from the Si substrate after 2 h, making the microrobots free-standing in the solution. Finally, we used DI water to clean the microrobots and transferred them to the swimming pool by the injector for the following magnetic driving experiment.

### Device simulation

The electromagnetic simulation software HFSS was used to simulate the device’s performance. The device was placed in the horizontal center of the simulation environment, and the excited port for generating the RF magnetic field was located above at least 6 times the device width to avoid the influence coming from the device being excited. In addition, the horizontal dimension was at least 4 times the device width so that the surrounding boundary was far enough away from the local area to avoid affecting the local RF magnetic field uniformity of the device. The simulation structure only included the three-layer structure of the sensor. To facilitate the calculation of the magnetic field integration, we directly defined the calculation plane with different $D$ values in the model to obtain the magnetic field distribution, whose size was consistent with the calculation plane. The integral of the magnetic field can be calculated by the HFSS, and simulation models of sensors with different structures were created. The corresponding response of the sensor to various conductivities and permittivities was simulated by changing the environmental dielectric properties.

### Device measurement

The PCB was connected to the network analyzer through an SMA connector. Considering the small size of the sensor, it was in direct contact with the PCB, leading to a close range between the sensor and the readout coil of ~10 µm. The covered epoxy resin not only fixed the chip on the PCB but also played a sealing role in protecting the solution from contacting the PCB from the back, resulting in parasitic capacitance. The hole in the center of the PCB can allow the sensor to soak in solution. The clean substrate without any structures was used as the control group. Solutions with different pH values were obtained by gradually diluting the purchased 0.1 mol/L HCl solution, and the corresponding pH value was confirmed by a pH meter. We used the arc funnel to inject the solution from the top, which can be discharged from the end under the immersed PCB. In contrast, to test the influence of different temperatures, the end of the PCB was placed in a beaker filled with DI water, whose temperature can be tuned by water-bath heating. The network analyzer was used for the frequency sweep under different environmental conditions.

### Supplementary information

Supporting information for

Linear and circular motion of a representative integrated AI microrobot with microsensor at 100 μm side length

Linear motion of a representative integrated AI microrobot with microsensor at 300 μm side length
