# Supplementary material for: Self-sensing intelligent microrobots for noninvasive and wireless monitoring systems
Source: Microsyst Nanoeng. 2023 Aug 9;9:102. doi: 10.1038/s41378-023-00574-4 (PMC10409863; doi:10.1038/s41378-023-00574-4)
Supplement: Supplementary file 1 — Supporting information for [file 41378_2023_574_MOESM1_ESM.docx]

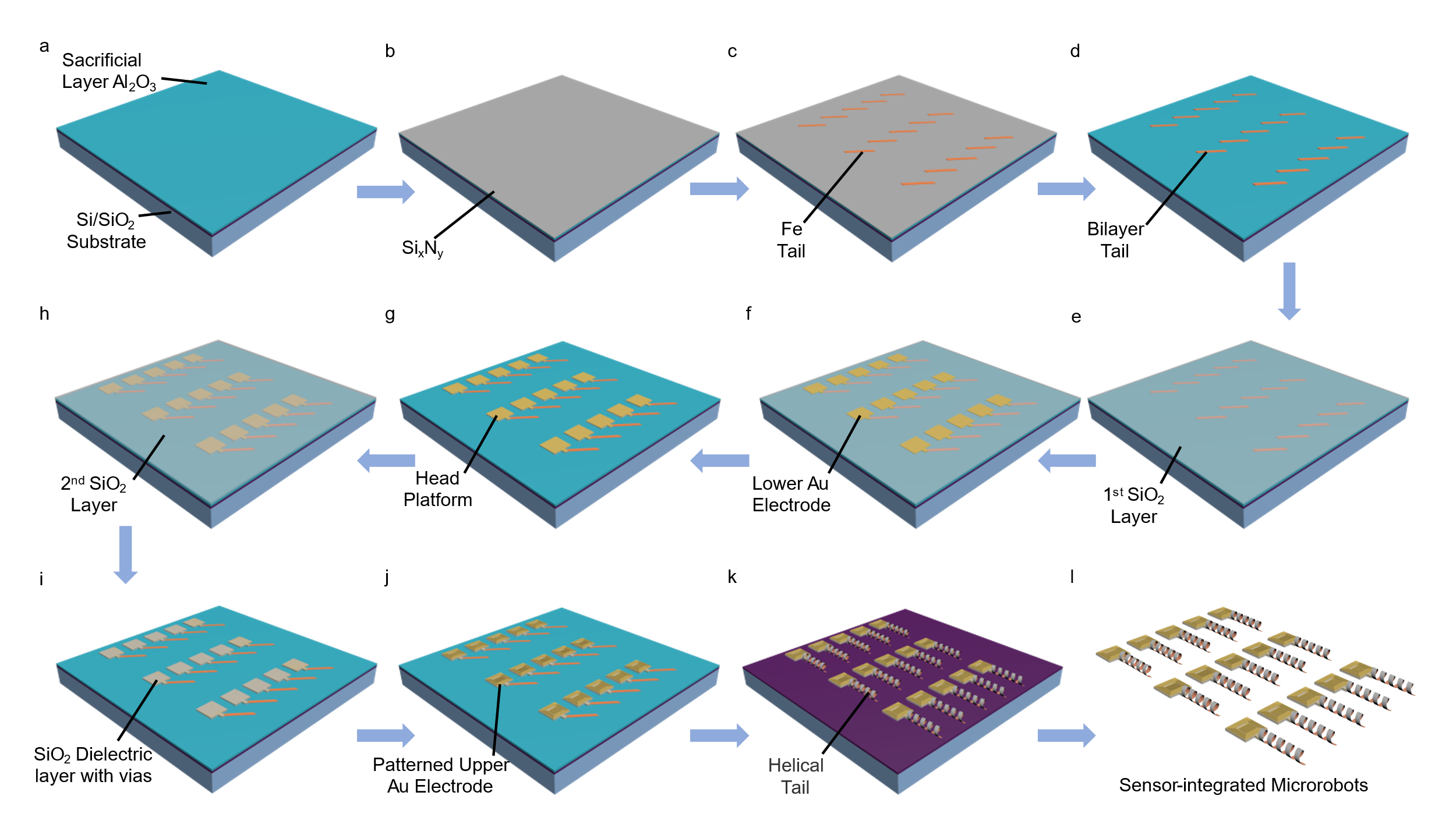


**Fig.S1. Fabrication processes for sensor-integrated microrobots. (a)** Evaporate 30 nm Al_2_O_3_ as a sacrificial layer using the e-beam. **(b)** Grow a Si_x_N_y_ pre-stressed film using PECVD. **(c)** Pattern Fe as the tail with a magnetic layer (Fe) using UV lithography and lift-off process. **(d)** Etch Si_x_N_y_ layer by ICP. **(e)** Evaporate 100 nm SiO_2_ using e-beam. **(f)** Pattern 100 nm Au as a lower electrode using UV lithography and lift-off process. **(g)** Etch the 1^st^ SiO_2_ layer by ICP. **(h)** Evaporate 100nm SiO_2_ using e-beam. **(i)** Etching the 2^nd^ SiO_2_ layer by RIE. **(j)** Pattern 300 nm Au as upper electrode using UV lithography and lift-off process. **(k)** Etch the Al_2_O_3_ layer to allow the bilayer tail to roll up. **(l)** The finished microrobots are released.


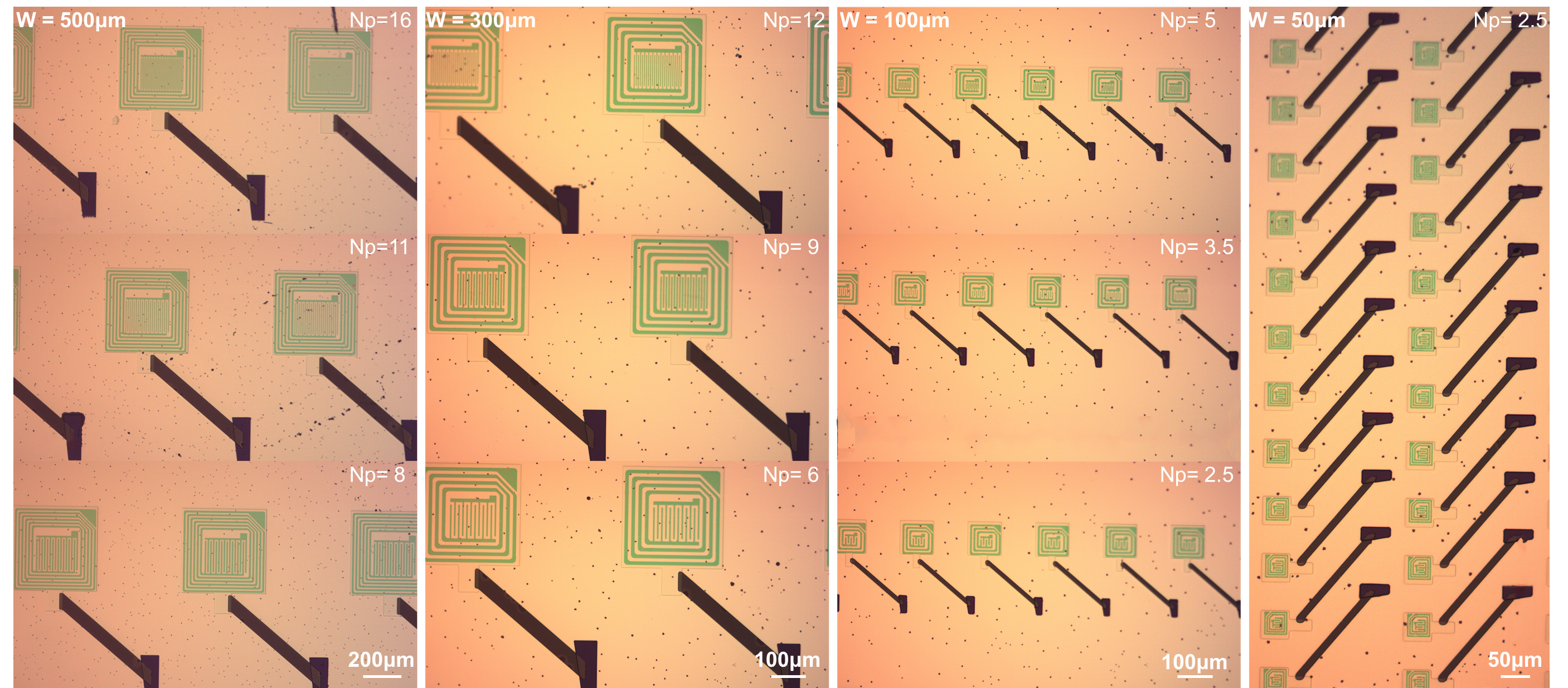


**Fig.S2. Microrobots with integrated sensors of different sizes and structures.** W: Width of the square substrate; Np: Number of interdigital electrode pairs. A certain distance is left around each structure to facilitate processing. About 25% of the area is used to place the interdigital electrodes. Each group has the same coil structure and variable interdigital electrode pairs.


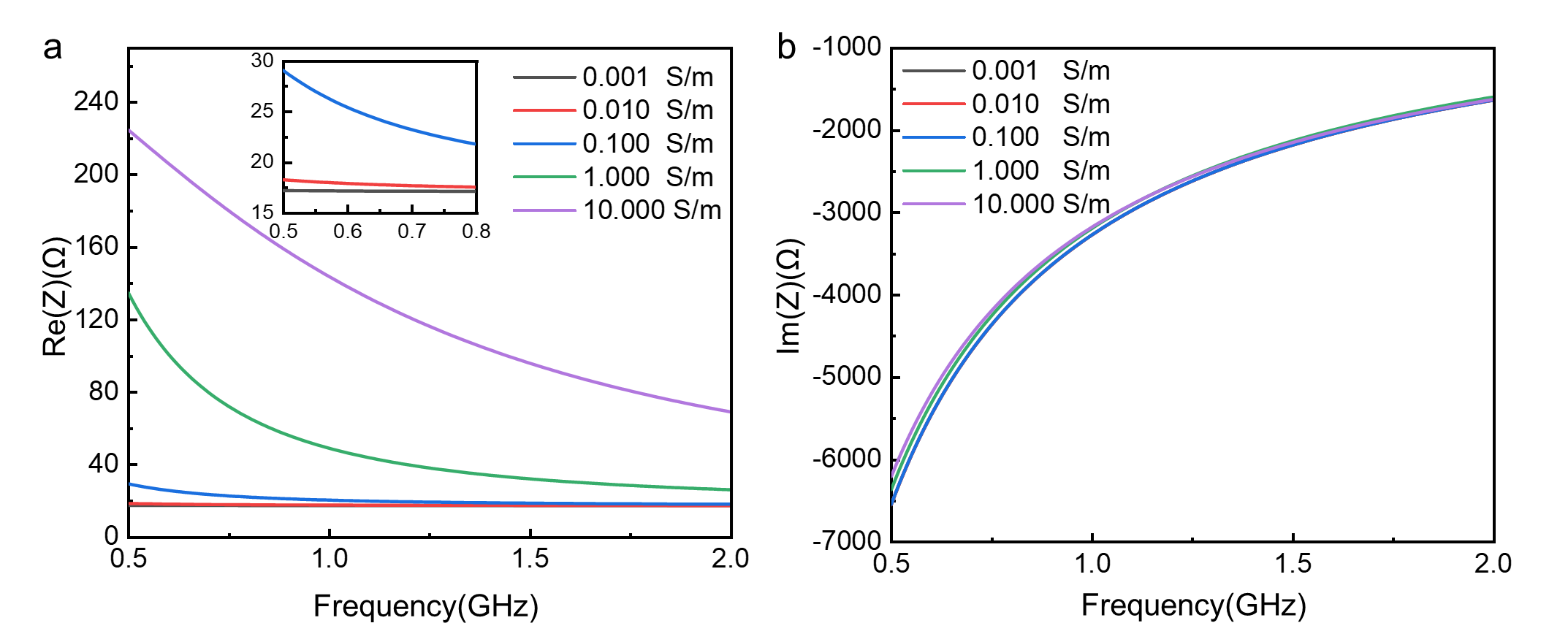


**Fig.S3. Simulation results of impedance sweep of interdigital electrodes depend on conductivity. (a), (b),** Real and imaginary parts of impedance as a function of the frequency with the environmental conductivity varying from 0.001 S/m to 10.000 S/m, respectively. The amplitudes of real part Re(Z) and imaginary part Im(Z) both show a downward trend with the increase in frequency. When conductivity increases, the real part’s amplitude increases, while the imaginary part’s amplitude decreases.


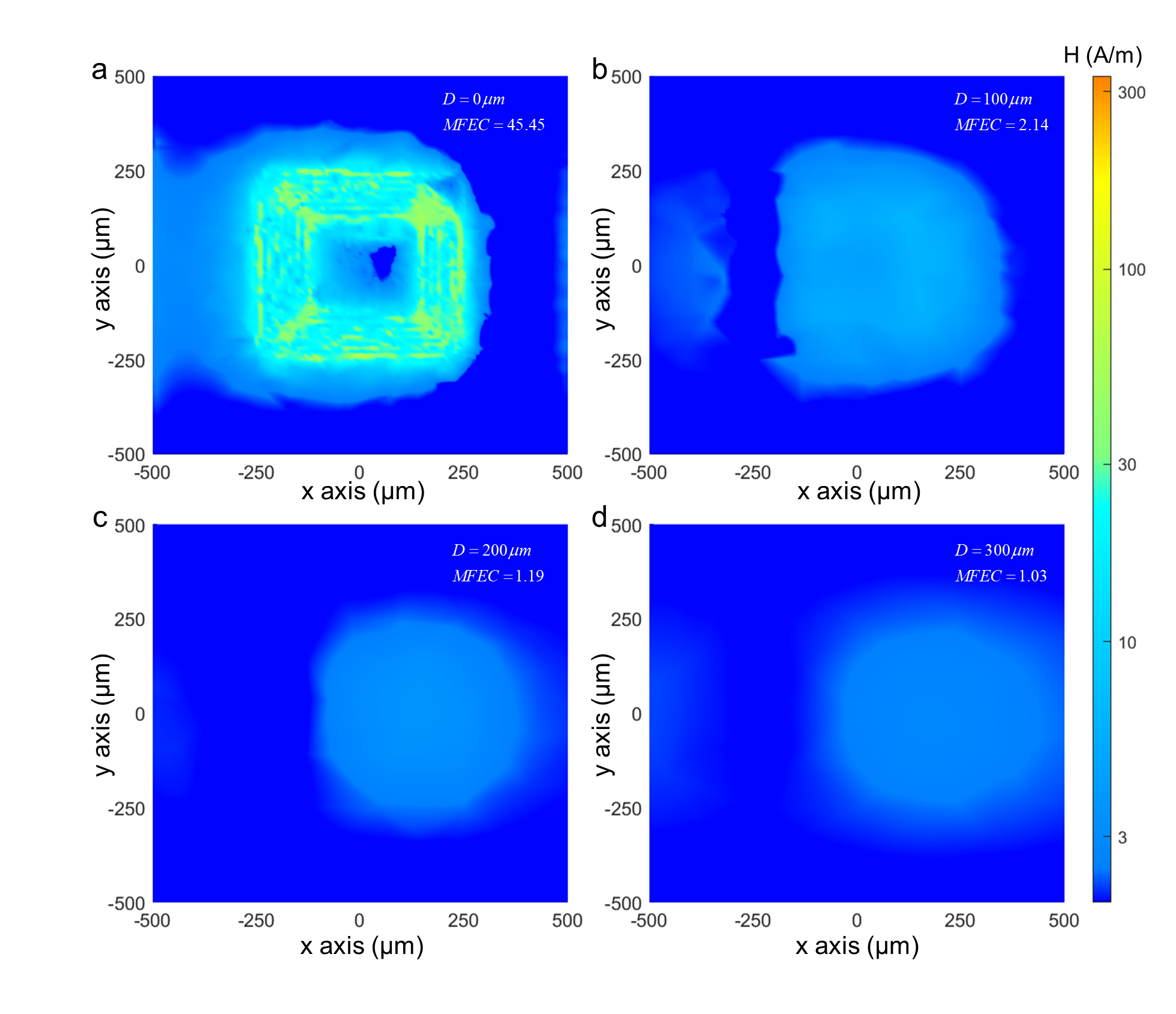


**Fig.S4. Electromagnetic field distribution of different heights D (0 μm, 100 μm, 200 μm, 300 μm) at the resonance point (f=1.24GHz) when ε_r_=81, σ=0 S/m.** The sensor enhances the local electromagnetic field and changes the distribution, but the enhancement effect gradually weakens with the increase of distance.


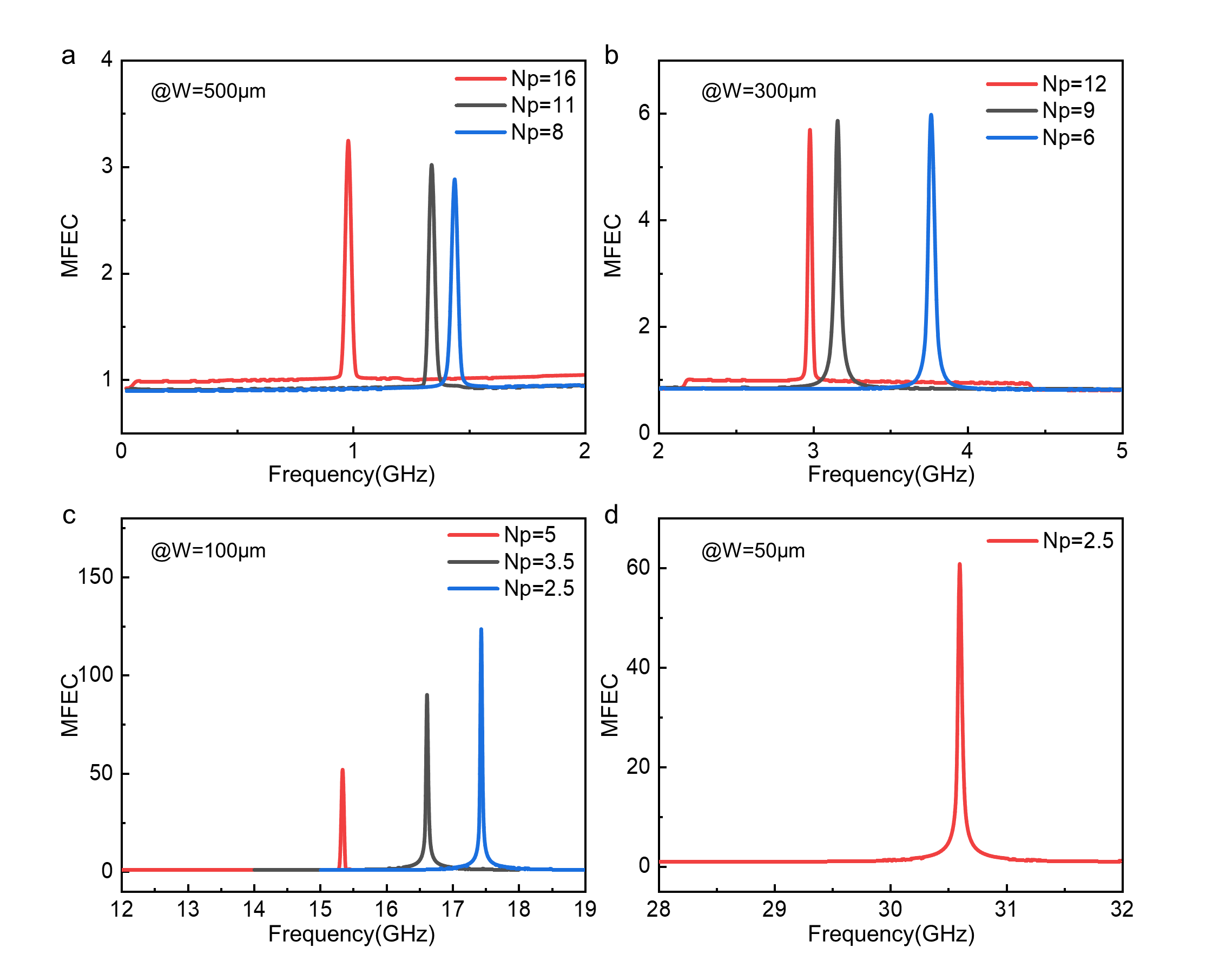


**Fig.S5. Frequency sweep simulation results of different sensors in deionized water.** Sensors with the same size have adjacent resonance peaks, but with the decrease in the sensor’s size, the resonance frequency increases from ~1GHz to ~30GHz. MFEC was calculated using the data at the substrate plane.


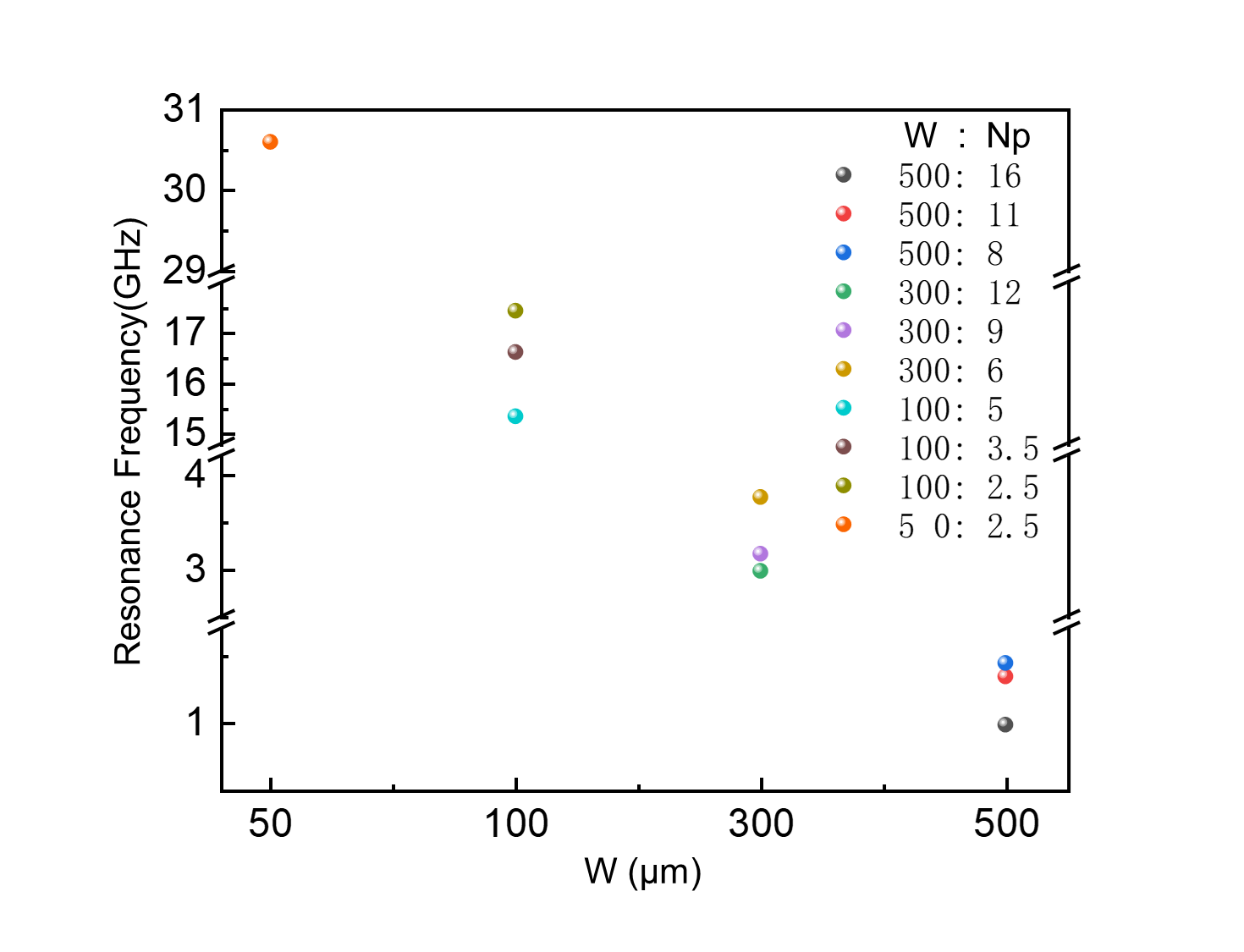


**Fig.S6. Resonance frequency of all sensors.** Resonance frequency decreases with the increase of the number of interdigital electrode pairs, but the sensor of bigger size always has a lower resonance frequency.


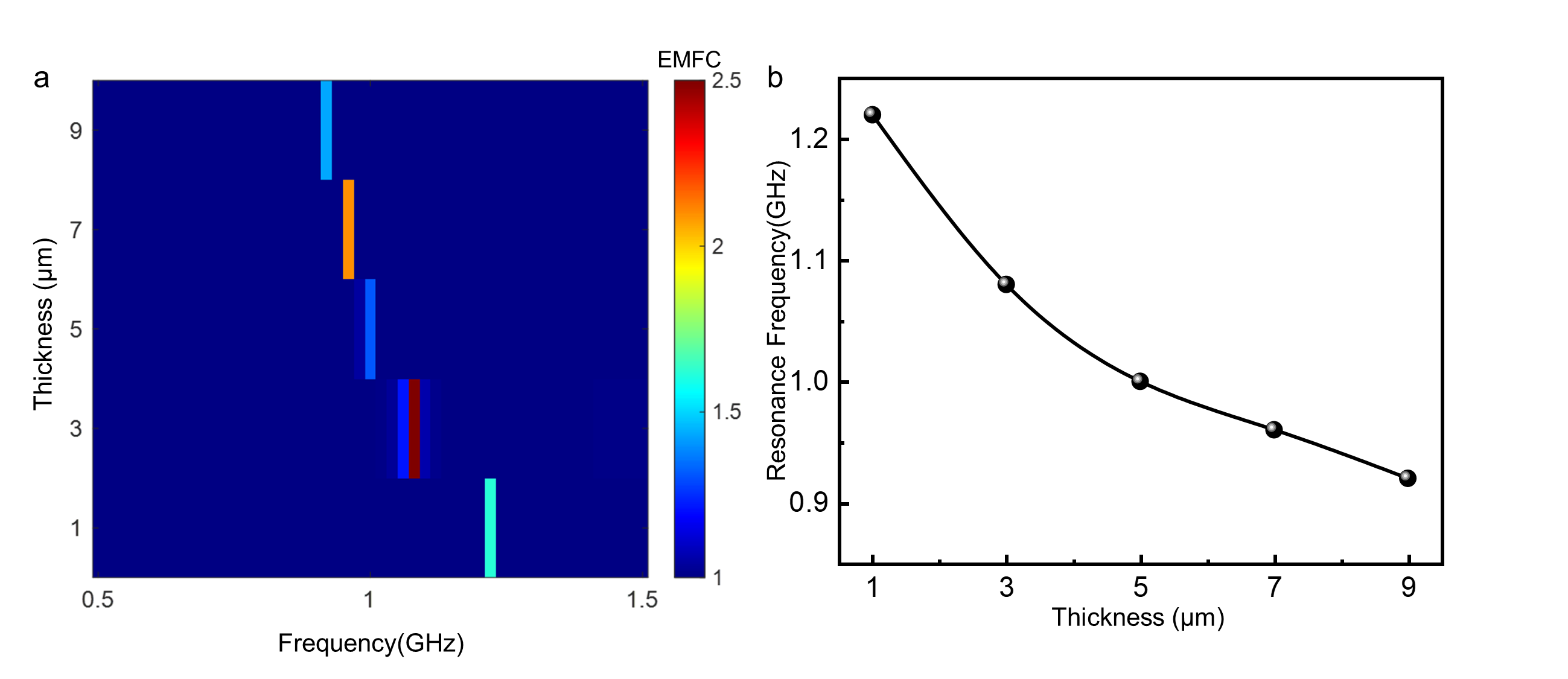


**Fig.S7. Sensor performance depends on electrode thickness. (a)** Sweeping frequency test of MFEC with varying thickness from 1 μm to 9 μm using the same sensor structure (W=500μm, Np=11). **(b)** Resonance frequency as a function of thickness. When thickness increases from 1μm to 9μm, resonance frequency decreases from 1.22 GHz to 0.92 GHz. The simulation was performed in deionized water and MFEC was calculated using the data at the substrate plane.


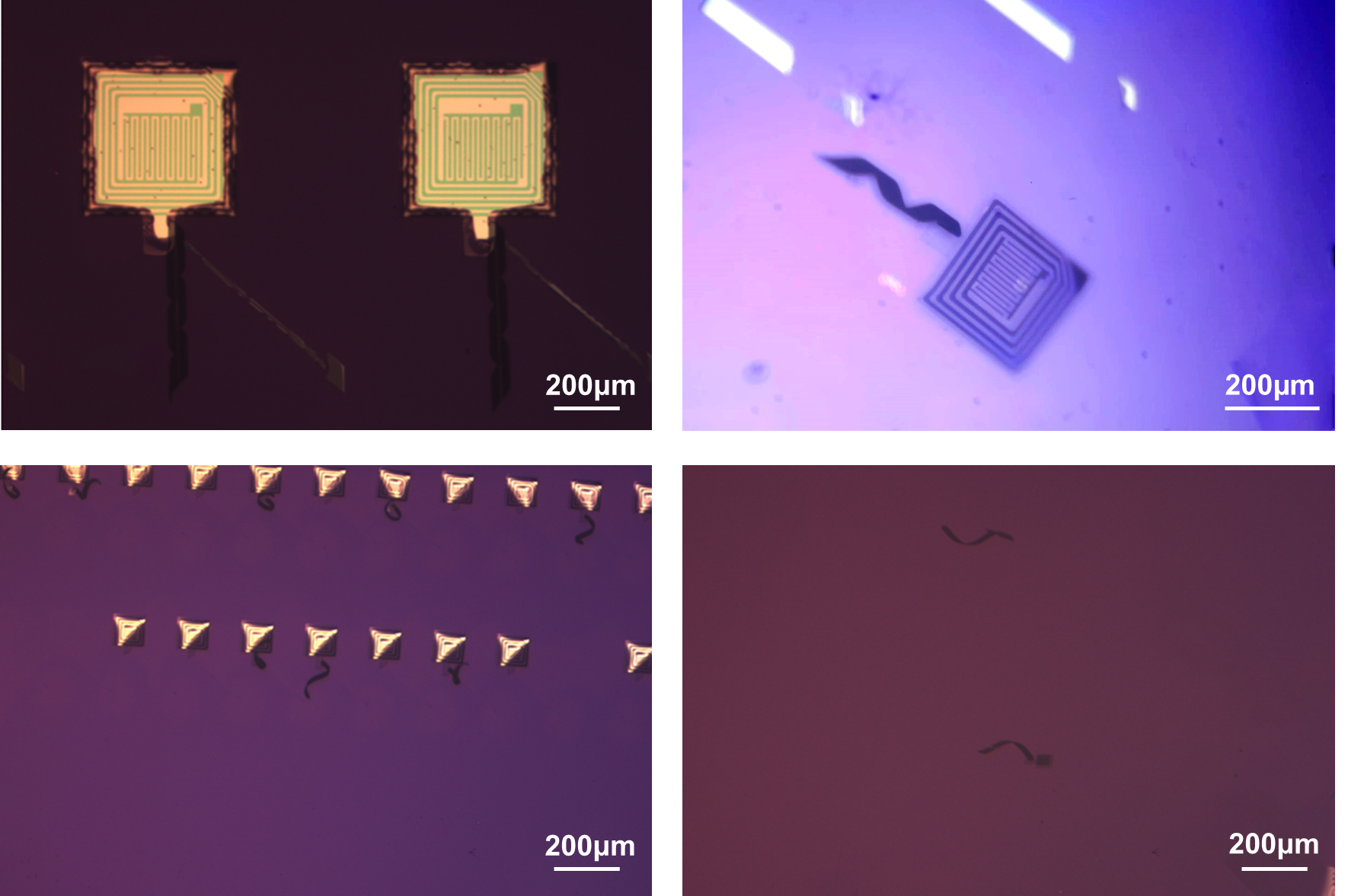


**Fig.S8. The integrated microrobots with different sizes were released into the liquid.** The microrobots swam in the liquid under the actuation of the external field.

**Supplementary Movie 1. Linear and circular motion of a representative integrated AI microrobot with microsensor at 100 μm side length.** The actuating magnetic induction intensity B_0_ = 6mT, and rotation frequency f_1_ = 1Hz, generating in the lateral direction. Then, a gradually increasing yaw angle with the increment speed at 24 °/s was applied to the initial field, forming the circular one. (The video was 1×).

**Supplementary Movie 2. Linear motion of a representative integrated AI microrobot with microsensor at 300 μm side length.** The actuating magnetic induction intensity B_0_ = 6mT, and rotation frequency f_1_ = 1Hz, generating in the lateral direction (The video was 1×).
